# Do Inter-Country Differences in the Frequency of Abusive Head Trauma Reflect Different Proportions of Overdiagnosis of Abuse or True Differences in Abuse?

**DOI:** 10.2188/jea.JE20190066

**Published:** 2020-06-05

**Authors:** Ulf Högberg, Waney Squier, Jacob Andersson, Göran Högberg, Vineta Fellman, Ingemar Thiblin, Knut Wester

**Affiliations:** 1 Department of Women’s and Children’s Health, Uppsala University, Uppsala, Sweden; 2 Department of Neuropathology, Oxford University John Radcliffe Hospital, Oxford, United Kingdom (Formerly; retired); 3 Forensic Medicine, Department of Surgical Sciences, Uppsala University, Uppsala, Sweden; 4 Department of Women’s and Children’s Health, Child and Adolescent Psychiatric Unit, Karolinska Institutet, Solna, Sweden (Formerly; retired); 5 Department of Clinical Sciences, Lund, Pediatrics, Lund University, Lund, Sweden; 6 Children’s Hospital, University of Helsinki and Folkhälsan Research Center, Helsinki, Finland; 7 Department of Clinical Medicine, University of Bergen and Department of Neurosurgery, Haukeland University Hospital, Bergen, Norway

The register study by Yamaoka et al examines presumptive (*N* = 324) and possible abusive head trauma (AHT) (*N* = 2,603) in infants up to 1 year of age derived from hospital discharge data based on the ICD-10 codes of head trauma, retinal haemorrhage, and intentional injuries, with exclusion of unintentional injury and fall accidents. They report an incidence of 7.2 per 100,000 and 41.7 per 100,000 for presumptive AHT and possible AHT respectively.^1^ Sweden had 2.3 per 100,000 infants born 1997–2014 with abuse diagnoses and subdural haemorrhage (SDH), including acute non-traumatic SDH,^2^ while the British Isles had 14.2 infants per 100,000 with SDH and abuse diagnosis during the years 1998–1999.^3^

One reason for the differences in incidence might be dissimilar diagnostic procedures between Japan, the British Isles, and Sweden. Yamaoka et al raise a limitation to their register study; that they did not have access to records, thus stating that the reported incidence might be an underestimate. However, Yamaoka et al do not question the possibility of overdiagnosis of abuse by failure to recognise natural conditions, such as Benign External Hydrocephalus (BEH).^4^ BEH is an apparently congenital condition that may be, and may have been, misdiagnosed as abuse. It is subtype of hydrocephalus, characterised by a rapid increase of head circumference (HC) in infancy, enlarged subarachnoid spaces (especially frontally), and normal or enlarged ventricles. Most of these infants are born with a normal HC that typically increases during the first months of life.^5^^,^^6^ Yamaoka et al are puzzled by their observation of two age peaks of incidence on “abuse”. While the first, at 1–3 months, coincides with the peak incidence of sudden infant death syndrome (SIDS), the second coincides with the mean age at referral of BEH.^5^ All three conditions (AHT, BEH, and SIDS) show a marked male preponderance and share many other demographic features.^2^^,^^5^^–^^7^ These similarities are indeed intriguing and may indicate causal relationships that deserve further exploration.

We question the validity of ICD codes in defining AHT: “an injury to the skull or intracranial contents of an infant or young child (<5 years of age) due to inflicted blunt impact and/or violent shaking”.^8^ A systematic literature review by the Swedish Agency for Health Technology Assessment and Assessment of Social Service identified circular reasoning as a major bias in AHT-diagnosis, and concluded that there “is insufficient scientific evidence on which to assess the diagnostic accuracy of the triad of subdural haemorrhage, retinal haemorrhage, and encephalopathy in identifying traumatic shaking (very low-quality evidence)”, or its components.^9^ Based upon the results of this review, the definition of AHT used by Yamaoka et al is unreliable. Although this systematic literature review has been much contested,^10^^–^^12^ it is a major step forward for evidence-based child protection. Overdiagnosis of infant abuse diagnosis has severe public health and ethical implications and threatens trust in child care.

We conclude that a major limitation of the provided incidences from Japan, the British Isles, and Sweden might be imprecise case definition of abuse, based on the presence of SDH, and not true differences in the incidence of abuse.

## References

[1] Yamaoka Y, Fujiwara T, Fujino Y, Matsuda S, Fushimi K Incidence and age distribution of hospitalized presumptive and possible abusive head trauma of children under 12 months old in Japan. J Epidemiol. 2020;30:91–97. 10.2188/jea.JE20180094 30713261 PMC6949182

[2] Högberg U, Andersson J, Squier W, Epidemiology of subdural haemorrhage during infancy: a population-based register study. PLoS One. 2018;13:e0206340. 10.1371/journal.pone.0206340 30379890 PMC6209227

[3] Hobbs C, Childs AM, Wynne J, Livingston J, Seal A Subdural haematoma and effusion in infancy: an epidemiological study. Arch Dis Child. 2005;90:952–955. 10.1136/adc.2003.037739 16113132 PMC1720567

[4] Wester K Two infant boys misdiagnosed as “shaken baby” and their twin sisters: a cautionary tale. Pediatr Neurol. 2019;97:3–11. 10.1016/j.pediatrneurol.2019.02.024 31147228

[5] Wiig US, Zahl SM, Egge A, Helseth E, Wester K Epidemiology of benign external hydrocephalus in Norway—a population-based study. Pediatr Neurol. 2017;73:36–41. 10.1016/j.pediatrneurol.2017.04.018 28666559

[6] Zahl SM, Egge A, Helseth E, Wester K Clinical, radiological, and demographic details of benign external hydrocephalus: a population-based study. Pediatr Neurol. 2019;96:53–57. 10.1016/j.pediatrneurol.2019.01.015 30808532

[7] Squier W, Mack J, Jansen AC Infants dying suddenly and unexpectedly share demographic features with infants who die with retinal and dural bleeding: a review of neural mechanisms. Dev Med Child Neurol. 2016 Dec;58(12):1223–1234. Epub 2016 Jul 20. 10.1111/dmcn.13202 27435495

[8] Parks S, Annest J, Hill H, Karch D. Pediatric Abusive Head Trauma: Recommended Definitions for Public Health Surveillance and Research. Centers for Disease Control and Prevention. https://www.cdc.gov/violenceprevention/pdf/pedheadtrauma-a.pdf. Published 2012. Accessed 6/12, 2014.

[9] Elinder G, Eriksson A, Hallberg B, Traumatic shaking: the role of the triad in medical investigations of suspected traumatic shaking. Acta Paediatr. 2018;107(Suppl 472):3–23. 10.1111/apa.14473 30146789 PMC6585638

[10] New Virtual Issue on Abusive Head Trauma and Shaken Baby Syndrome. Acta Paediatr. First published 23 january 2019. https://onlinelibrary.wiley.com/doi/toc/10.1111/(ISSN)1651-2227.Abusive-Head-Trauma?campaign=dartwol|4933896936.

[11] Debelle GD, Maguire S, Watts P, Nieto Hernandez R, Kemp AM; Child Protection Standing Committee, Royal College of Paediatrics and Child Health Abusive head trauma and the triad: a critique on behalf of RCPCH of ‘Traumatic shaking: the role of the triad in medical investigations of suspected traumatic shaking’. Arch Dis Child. 2018;103(6):606–610. 10.1136/archdischild-2017-313855 29510999

[12] Lynøe N, Elinder G, Hallberg B, Rosén M, Sundgren P, Eriksson A Easier to see the speck in your critical peers’ eyes than the log in your own? Response to Debelle *et al*. Arch Dis Child. 2018;103(7):714. 29728419 10.1136/archdischild-2018-315380

